# Individual and Contextual Factors Associated with Low Childhood Immunisation Coverage in Sub-Saharan Africa: A Multilevel Analysis

**DOI:** 10.1371/journal.pone.0037905

**Published:** 2012-05-25

**Authors:** Charles S. Wiysonge, Olalekan A. Uthman, Peter M. Ndumbe, Gregory D. Hussey

**Affiliations:** 1 Vaccines for Africa Initiative, Institute of Infectious Disease and Molecular Medicine, University of Cape Town, Cape Town, South Africa; 2 Division of Medical Microbiology, Department of Clinical Laboratory Sciences, University of Cape Town, Cape Town, South Africa; 3 African Public Health Foundation, Abuja, Nigeria; 4 Primary Care Sciences, Keele University, Staffordshire, United Kingdom; 5 Faculty of Health Sciences, University of Buea, Buea, Cameroon; University of Massachusetts Medical School, United States of America

## Abstract

**Background:**

In 2010, more than six million children in sub-Saharan Africa did not receive the full series of three doses of the diphtheria-tetanus-pertussis vaccine by one year of age. An evidence-based approach to addressing this burden of un-immunised children requires accurate knowledge of the underlying factors. We therefore developed and tested a model of childhood immunisation that includes individual, community and country-level characteristics.

**Method and Findings:**

We conducted multilevel logistic regression analysis of Demographic and Health Survey data for 27,094 children aged 12–23 months, nested within 8,546 communities from 24 countries in sub-Saharan Africa. According to the intra-country and intra-community correlation coefficient implied by the estimated intercept component variance, 21% and 32% of the variance in unimmunised children were attributable to country- and community-level factors respectively. Children born to mothers (OR 1.35, 95%CI 1.18 to 1.53) and fathers (OR 1.13, 95%CI 1.12 to 1.40) with no formal education were more likely to be unimmunised than those born to parents with secondary or higher education. Children from the poorest households were 36% more likely to be unimmunised than counterparts from the richest households. Maternal access to media significantly reduced the odds of children being unimmunised (OR 0.94, 95%CI 0.94 to 0.99). Mothers with health seeking behaviours were less likely to have unimmunised children (OR 0.56, 95%CI 0.54 to 0.58). However, children from urban areas (OR 1.12, 95% CI 1.01 to 1.23), communities with high illiteracy rates (OR 1.13, 95% CI 1.05 to 1.23), and countries with high fertility rates (OR 4.43, 95% CI 1.04 to 18.92) were more likely to be unimmunised.

**Conclusion:**

We found that individual and contextual factors were associated with childhood immunisation, suggesting that public health programmes designed to improve coverage of childhood immunisation should address people, and the communities and societies in which they live.

## Introduction

The 2015 deadline for achievement of the Millennium Development Goals (MDGs) is less than five years away, and Africa is significantly behind the rest of the world in making good its commitment to reduce child mortality by two-thirds [1]. Africa has the highest under-five mortality rate of all the world’s continents, with 40% of all global deaths in under five year olds occurring in African countries located south of the Sahara desert. Globally, the under-five mortality rate has decreased by 26% from 91 deaths per 1000 live births in 1990 to 67 deaths per 1000 live births in 2007; while in sub-Saharan Africa the rate has fallen by only 20%, from 181 to 145 over the same period. Vaccine-preventable diseases are a major contributor to high African child mortality rates, partly because of the limited introduction of new vaccines and low uptake of existing vaccines. At present, only 71% of African infants receive the full series of three doses of the diphtheria-tetanus-pertussis vaccine (DTP3). There is wide inter-country variation in reported DTP3 coverage, from 23% in Chad to 99% in Mauritius [2]. Overall more than six million children in sub-Saharan Africa did not receive DTP3 by one year of age in 2010. Vaccine efficacy tends to be lower in low-income countries than in higher-income countries [3], [4], emphasising the need to attain and sustain high and equitable childhood immunisation coverage in sub-Saharan Africa; where most countries are low-income.

As sub-Saharan Africa continues to grapple with a range of programme and policy challenges related to childhood immunisation, we believe that one important element in improving the status quo is a comprehensive and relevant evidence base that would equip countries in the region to take informed actions. Without comprehensive information about the factors associated with failure to complete the full series of recommended vaccines, it is hard to plan substantial public health programmes that would improve childhood immunisation programmes in the region. Numerous studies have been conducted to examine factors associated with childhood immunisation in sub-Saharan Africa [5], [6], [7], [8], [9], [10], [11], [12], [13]. Preponderance of these studies has concentrated on individual-level factors [5], [6], [7], [8], [9], [10], [11] and only few have considered community-level factors [5], [12], [13]. To the best of our knowledge, there has been no multilevel study performed to date that examined the separate and independent contributions of individual, community, and country-level factors to the low uptake of immunisation services in sub-Saharan Africa. We therefore conducted this study to fill this research gap and to draw attention to the largely unexplored contextual factors that may be associated with low childhood immunisation coverage. The objective of this study was therefore to develop and test a model of childhood immunisation that includes individual-level characteristics along with contextual characteristics defined at the community and country levels.

## Methods

### Ethics Statement

We based this study on an analysis of existing survey data collected by the Monitoring and Evaluation to Assess and Use Results Demographic and Health Surveys (MEASURE DHS) project (www.measuredhs.com). Since 1984, the MEASURE DHS project has collected standardised nationally representative survey data in over 90 countries [14]. The surveys included in this study were approved by the Institutional Review Board of Macro International in Calverton in the United States of America and by the National Ethical Review Committees in Benin, Burkina Faso, Cameroon, Chad, Democratic Republic of Congo, Ethiopia, Ghana, Guinea, Kenya, Lesotho, Madagascar, Malawi, Mali, Namibia, Niger, Nigeria, Rwanda, Sao Tome & Principle, Senegal, Sierra Leone, Swaziland, Uganda, Zambia, and Zimbabwe. All study participants gave written informed consent before participation and all information was collected confidentially. We obtained the raw survey data and written consent of MEASURE DHS to use the data.

### Data Source

We used data available as of November 2011 from 24 Demographic and Health Surveys (DHS) conducted by the MEASURE DHS project between 2003 and 2010 in sub-Saharan Africa. Methods and data collection procedures have been published elsewhere [14]. Briefly, DHS surveys are implemented by respective national institutions and Macro International Inc., with financial support from the US Agency for International Development (USAID). Selection of the countries in this study was determined by availability of comparable data on childhood immunisation. DHS data are nationally representative, cross-sectional, household sample surveys with large sample sizes, typically between 5,000 and 15,000 households. The sampling design typically involves selecting and interviewing separately nationally representative probability samples of women aged 15–49 years and men aged 15–59 years based on multi-stage cluster sampling, using strata for rural and urban areas and for different regions of the countries. A standardized questionnaire was administered by interviewers to participants in each country. The survey instruments (i.e. household questionnaire and women’s questionnaire) were comparable across countries, yielding inter-country comparable data. We used the term community to describe clustering within the same geographical living environment. Communities were based on sharing a common primary sample unit (PSU) within the DHS data. The sampling frame for identifying PSU in the DHS is usually the most recent census. Country-level data were collected from the reports published by the World Bank [15].

### Outcome Variable

We used the World Health Organisation (WHO) definition of an “unimmunised child” as the outcome variable. “Unimmunised child” was defined as a binary variable that takes the value of 1 if the child 12–23 months old has received DTP3 and 0, otherwise. We limited the analysis to one child per woman in order to minimise over-representation of women with more than one child in the age category.

### Determinant Variables

#### Individual-level

We included the following individual level factors: child’s age (in months), child sex (male or female), high birth order (less than 24 months), number of under-five children, polygamous family, mother’s age (15–24, 25–34, or 35 years or older), wealth index (poorest, poorer, middle, richer, richest), mother’s and father’s education (no education, primary, secondary, or higher), employment status (working or not working), media access (access to radio, television or newspaper), and maternal health seeking behaviours (prenatal visits, tetanus injection during pregnancy, medical assistance at delivery, knowledge of oral rehydration solution (ORS), and possession of a health card for the child).

#### Community-level

We included the following community-level factors:

1. *Neighbourhood poverty:* percentage of households below 20% of wealth index
2. *Illiteracy rate*: percentage of women with no formal education in the community
3. *Unemployment rate*: percentage of women not working in the community
4. *Media access*: percentage of households with access to television, radio or newspaper
5. *Average household size:* mean number of people in each community
6. *Female-headed households*: percentage of households headed by women in an area.
7. *Residential mobility:* proportion of households occupied by persons who had moved from another dwelling in the previous 5 years [16], [17], [18]
8. *Place of residence:* urban or rural, as administratively defined by each country
9. *Ethnic diversity -* an index of ethnic diversity was created using a formula that captures both the number of different groups in an area and the relative representation of each group [19]:

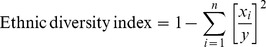
where:


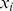
 = population of ethnic group *i* of the area,

*y*  =  total population of the area, and

*n*  =  number of ethnic groups in the area.

Scores can range from 0 to approximately 1. For clarity of interpretation, each diversity index is multiplied by 100; the larger the index, the greater diversity there is in the area. If an area’s entire population belongs to one ethnic group, then an area has zero diversity. An area’s diversity index increases to 100 when the population is evenly divided into ethnic groups.

#### Country-level

At country-level, we included fertility rate, gross domestic product (GDP), expenditure on health, and adult illiteracy rate. We categorised community- and country-level variables into two categories (low and high) to allow for nonlinear effects and provide results that were more readily interpretable in the policy arena. Median values served as the reference group for comparison.

### Control Variable

The year the DHS was conducted was included as a partial control for a period trend to control for effects of unknown factors that may have been introduced due to different timing of surveys across countries.

### Statistical Analyses

Multilevel logistic regression models were used to examine factors associated with childhood immunisation. We specified a 3-level model for the binary variable: an “unimmunised child” (level 1), living in a community (level 2), from a country (level 3). We constructed six models. The first model, an empty model, was without any determinant variables i.e. a simple component of variance analysis. The second model contained only the control variable (survey year). The third, fourth, and fifth models provided additional controls for individual-, community- and country-level factors respectively. The sixth model simultaneously controlled for survey year, individual-, community-, and country-level factors.

The measures of association (fixed-effects) were reported as odds ratios (ORs) with their 95% confidence intervals (CIs). The measures of variation (random-effects) included variance, intra-cluster correlation (ICC), and median odds ratio (MOR). The ICC was calculated by the linear threshold according to the formula used by Snijders and Bosker [20]. MOR is a measure of unexplained cluster heterogeneity and the method used for calculating MOR has been described elsewhere [21], [22]. The multilevel models were fitted with MLwiN 2.24 [23]. The statistical significance of covariates were calculated using the Wald test [23]. All significance tests were two-tailed and statistical significance was defined at the 5% alpha level.

## Results

### Sample Characteristics

**Figure 1:**
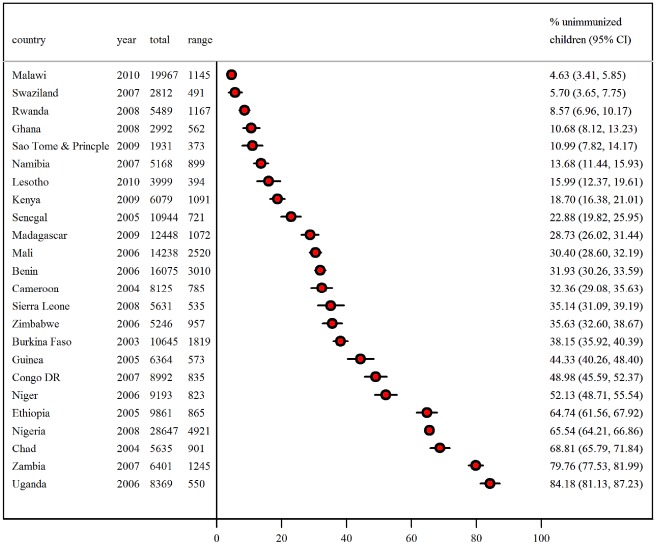
Description of Demographic and Health Survey data 2003–2010 in sub-Saharan Africa by country, survey year, sample size, and percentage of unimmunised children aged 12–23 months.

The survey characteristics are shown in Figure 1. The DHS were conducted between 2003 and 2010. The number of children included in the surveys ranged from 1,931 in Sao Tome and Principe to 28,647 in Nigeria. The age of children included in the analysis ranged from 12 to 23 months. The median number of children is 882 (range: 373 to 4921). The proportion of unimmunised children ranges from as low as 4.6% in Malawi to as much as 84.2% in Uganda.

### Measures of Variation (Random-effects)

As shown in Table 1, Model 1 (the null model), there was a significant variation in the odds of being an “unimmunised child” across the countries (*τ*  = 1.031, p<.001) and across the communities (*τ*  = 0.554, p<.01). According to the intra-country and intra-community correlation coefficient implied by the estimated intercept component variance, 21% and 32% of the variance in the “unimmunised child” could be attributed to the country- and community-level factors respectively. The variations across communities and countries remained statistically significant, even after controlling for all factors in model 6.

**Table 1:** Factors associated with unimmunised children in sub-Saharan Africa, identified by multilevel logistic regression models.

	Model 1^a^	Model 2^b^	Model 3^c^	Model 4^d^	Model 5^e^	Model 6^f^
Factors	OR (95% CI)	OR (95% CI)	OR (95% CI)	OR (95% CI)	OR (95% CI)	OR (95% CI)
Control variable						
Survey year						
2003		1 (reference)	1 (reference)	1 (reference)	1 (reference)	1 (reference)
2004		1.70 (0.21–13.89)	1.84 (0.26–12.80)	1.82 (0.20–16.58)	0.42 (0.04–4.74)	0.31 (0.03–2.98)
2005		1.26 (0.17–9.13)	1.11 (0.18–6.90)	1.25 (0.16–10.04)	1.47 (0.19–11.38)	0.98 (0.15–6.46)
2006		1.44 (0.22–9.39)	1.88 (0.33–10.67)	1.53 (0.21–11.05)	0.53 (0.07–4.15)	0.50 (0.08–3.10)
2007		0.96 (0.14–6.57)	1.73 (0.29–10.19)	0.98 (0.13–7.34)	0.59 (0.07–5.24)	0.54 (0.08–3.81)
2008		0.67 (0.10–4.54)	1.02 (0.16–6.33)	0.66 (0.09–4.99)	1.53 (0.15–15.87)	1.04 (0.13–8.38)
2009		0.39 (0.05–2.85)	0.61 (0.10–3.79)	0.39 (0.05–3.10)	0.22 (0.03–1.64)	0.24 (0.04–1.42)
2010		0.18 (0.02–1.48)	0.29 (0.04–2.05)	0.20 (0.02–1.81)	0.19 (0.02–1.92)	0.17 (0.02–1.36)
Child’s age			0.97 (0.96–0.98)***			0.97 (0.96–0.98)***
Male (vs. female)			1.01 (0.95–1.08)			1.01 (0.95–1.07)
High birth order			1.05 (0.96–1.14)			1.05 (0.97–1.14)
Under-5 children			1.02 (0.99–1.05)			1.02 (0.99–1.05)
Polygamous family			1.08 (1.01–1.16)			1.08 (1.01–1.16)*
Mother’s age						
15–24			1.22 (1.09–1.36)***			1.18 (1.06–1.32)**
25–34			1.07 (0.98–1.16)			1.05 (0.96–1.15)
35 or older			1 (reference)			1 (reference)
Wealth index						
Poorest			1.34 (1.17–1.52)***			1.36 (1.17–1.59)***
Poorer			1.25 (1.11–1.42)***			1.30 (1.13–1.51)***
Middle			1.14 (1.01–1.29)*			1.21 (1.06–1.39)**
Richer			1.09 (0.97–1.23)			1.15 (1.02–1.30)*
Richest			1 (reference)			1 (reference)
Mother’s education						
No education			1.48 (1.31–1.67)***			1.35 (1.18–1.53)***
Primary			1.28 (1.15–1.43)***			1.26 (1.12–1.40)***
Secondary or higher			1 (reference)			1 (reference)
Father’s education						
No education			1.19 (1.07–1.31)**			1.13 (1.02–1.26)*
Primary			0.99 (0.90–1.09)			1.00 (0.91–1.10)
Secondary or higher			1 (reference)			1 (reference)
Not working			1.09 (1.01–1.17)*			1.06 (0.98–1.14)
Media access			0.94 (0.90–0.98)**			0.94 (0.90–0.99)*
Health seeking beh.			0.54 (0.53–0.56)***			0.56 (0.54–0.58)***
COMMUNITY-LEVEL						
Urban (vs. rural)				0.84 (0.77–0.92)***		1.12 (1.01–1.23)*
Ethnic diversity				1.05 (0.97–1.13)		1.08 (1.00–1.16)
Neigh. Poverty				1.24 (1.15–1.34)***		1.03 (0.94–1.13)
% female-headed				0.88 (0.82–0.93)***		0.95 (0.89–1.02)
Residential instability				0.98 (0.92–1.05)		1.02 (0.95–1.09)
Illiteracy rate				1.62 (1.50–1.74)***		1.13 (1.05–1.23)**
Unemployment rate				1.14 (1.07–1.22)***		1.03 (0.96–1.11)
% media access				0.71 (0.66–0.77)***		0.94 (0.86–1.02)
Av. Household size				1.07 (1.00–1.14)		1.02 (0.95–1.09)
COUNTRY-LEVEL						
Fertility rate					4.47 (1.19–16.74)*	4.43 (1.04–18.92)*
GDP (US$)					1.75 (0.48–6.31)	1.44 (0.33–6.37)
Health expenditure					0.37 (0.11–1.23)	0.62 (0.19–2.03)
Literacy rate					2.25 (0.71–7.07)	2.60 (0.90–7.50)
RANDOM PART						
Variance						
Country	1.031 (0.300)	0.761 (0.222)	0.648 (0.194)	0.842 (0.246)	0.631 (0.185)	0.492 (0.148)
Community	0.554 (0.029)	0.663 (0.032)	0.275 (0.029)	0.440 (0.028)	0.751 (0.035)	0.267 (0.028)
ICC (%)						
Country	21.1	16.1	15.4	18.4	13.5	12.1
Community	32.5	30.2	21.9	28.0	29.6	18.7
MOR						
Country	2.62	2.29	2.15	2.39	2.13	1.95
Community	3.31	3.11	2.49	2.93	3.06	2.89

* p<0.01, **p<0.001 ***p<0.0001; ICC – intra-cluster correlation; MOR – median odds ratio; OR- odds ratio; CI – confidence interval.

a Model 1 is null model, baseline model without any determinant variable.

b Model 2 is adjusted for control variable alone.

c Model 3 is additionally adjusted for control variable and individual-level factors.

d Model 4 is additionally adjusted for control variable and community-level factors.

e Model 5 is additionally adjusted for control variable and country-level factors.

f Model 6 is additionally adjusted for control variable and individual-, community-, and country-level factors.

Results from the MOR also confirmed evidence of community and country contextual phenomena shaping the odds of being an “unimmunised child”. The high MOR (3.31) in Model 1 between children with a higher and lower propensity of being “unimmunised” in a community suggests that the community heterogeneity is substantial. Including all factors reduced the unexplained heterogeneity between communities to an MOR of 2.29.

### Measures of Association (Fixed Effects)

The results of fitting the model including only the control variable (survey) is shown in Table 1 (model 2). There was no statistically significant association between the survey year and the odds of being unimmunised. The results of fitting the model including the control variable (survey) and individual-level factors is shown in Table 1 (model 3). For every one month increase in a child’s age, the odds of being unimmunised decreased by 3% (odds ratio [OR] 0.97, 95% confidence interval [CI] 0.96 to 0.98). Compared with children of older mothers (i.e. 35 years or older), children of younger mothers were more likely to be unimmunised (OR 1.22, 95% CI 1.09 to 1.36). Similarly, children from the poorest households were more likely to be unimmunised than their counterparts from the richest households. Children born to mothers (OR 1.48, 95% CI 1.31 to 1.67) or fathers (OR 1.19, 95% CI 1.07 to 1.31) with no formal education were more likely to be unimmunised than those born to parents with secondary or higher education respectively. Children whose mothers were unemployed were more likely to be unimmunised than those whose mothers were employed (OR 1.09, 95% CI 1.01 to 1.17). Maternal access to media reduced the odds of a child being unimmunised by 6% (OR 0.94, 95% CI 0.94 to 0.98). Mothers with health seeking behaviours were 46% less likely to have children that were unimmunised (OR 0.54, 95% CI 0.53 to 0.56).

The results of fitting the model including the control variable (survey) and community-level factors is shown in Table 1 (model 4). Children from urban areas were less likely to be unimmunised than those from rural areas (OR 0.84, 95% CI 0.77 to 0.92). Children from communities with high neighbourhood poverty (OR 1.24, 95% CI 1.15 to 1.34), illiteracy (OR 1.62, 95% CI 1.50 to 1.74), and unemployment (OR 1.14, 95% CI 1.07 to 1.22) rates were 24%, 62% and 14% more likely to be unimmunised. Children from communities with high media access (OR 0.71, 95% CI 0.66 to 0.77) and female-headed households (OR 0.88, 95% CI 0.82 to 0.93) were 29% and 12% less likely to be unimmunised.

The results of fitting the model including the control variable (survey) and country-level factors is shown in Table 1 (model 5). Children from countries with higher fertility rates were more than four times more likely to be unimmunised.

The result of the full model including all co-variables is shown in Table 1 (model 6). With all factors controlled for statistically, the following factors remained significantly associated with the odds of being unimmunised: individual-level (child’s age, polygamous family, mother’s age, wealth index, mother’s and father’s education, media access, and maternal health seeking behaviours); community-level (place of residence and illiteracy rate); and country-level (fertility rate).

## Discussion

### Summary of Findings

The present study expands upon previous literature in providing evidence of contextual factors, measured at community and country levels, associated with childhood immunisation coverage. In particular, the study provided evidence that unimmunised children were more likely to be from communities with high illiteracy rates and countries with high fertility rates. Contrary to expectation, we found children from urban areas were more likely to be unimmunised than those from rural areas. At individual level, children from poorest households, uneducated parents, mothers with no access to media, and mothers with low health seeking behaviours were more likely to be unimmunised. In addition, we found evidence of clustering effects of non-immunisation at both community and country levels, such that children from the same communities tended to have similar immunisation status. This suggests that children in the same neighbourhood are subject to common contextual influences [24]; thus, providing evidence of contextual phenomenon shaping children’s risk of being unimmunised.

### Implication of the Findings

An evidence-informed approach to improving childhood immunisation programmes in sub-Saharan Africa and, consequently, reducing child mortality in the region demands first an understanding of why programmes have failed to reach their optimum potential. Secondly, appropriate strategies should be employed to address the identified short comings of immunisation programmes. Without such a systematic approach, significant proportions of scarce resources will continue to be squandered on ineffective interventions; and millions of children will continue to die from easily preventable causes [25], [26].

Our multilevel analysis reveal that low parental and community knowledge of immunisation and/or lack of access to information on childhood immunisation could be an important contributor to the high burden of unimmunised children in sub-Saharan Africa. This assertion is supported by the finding of significant reductions in the number of unimmunised children among parents and communities with access to mass media. In addition, unimmunised children were found to cluster in communities, increasing the risk of disease outbreaks. It is therefore important to identify effective interventions to enable parents and communities to understand the meaning and relevance of vaccination to their health and the health of their families and communities. However, at present, there is a paucity of synthesised research evidence on effective interventions for improving childhood immunisation programmes in low and middle-income settings such as sub-Saharan Africa [27], [28], [29], [30], [31].

The few currently available systematic reviews relevant to childhood immunisation programmes in sub-Saharan Africa show that parent reminder and recall systems [32] and mass media interventions [33] have the potential to increase immunisation coverage. Verbal, video, or provider-delivered communication tools may also increase parents’ understanding, especially if the tools are structured, tailored and interactive [34]. In addition, interventions to promote interaction between the community and health services may build trust and generate awareness and understanding of vaccination issues among parents [28]. Interventions of this nature embrace collective decision making and community involvement in planning, programme delivery, advocacy, and/or governance of immunisation programmes. The end result would be an increased demand for immunisation services [35].

The finding that women with health seeking behaviours are more likely to have their children immunised might be an indication that integration is an effective strategy in immunisation programmes. While there is limited data showing that childhood immunisation programmes could effectively be used as vehicles for other child survival interventions such as insecticide treated nets, vitamin A and deworming tablets [35], [36], we should guard against “milking the willing horse to death.” In this regard, others have cautioned that “where immunisation performance is strong, immunisation contacts may be excellent vehicles for additional interventions…but where an immunisation service is struggling, adding another child survival intervention on to immunisation might be the straw that breaks its back” [36]. This is an indication that immunisation programmes need to have an integral robust monitoring and evaluation framework in order to inform decisions being made.

### Conclusion

We found that individual and contextual factors were associated with childhood immunisation, suggesting that public health programmes designed to improve childhood immunisation coverage in sub-Saharan Africa should address people and the communities and societies in which they live. Synthesis of existing immunisation barriers and the evidence on effective interventions to address these barriers should be a systematic and integral component of childhood immunisation programmes in Africa, and elsewhere.
